# Different Sources of Omega-3 Fatty Acid Supplementation vs. Blood Lipid Profiles—A Study on a Rat Model

**DOI:** 10.3390/foods13030385

**Published:** 2024-01-24

**Authors:** Ewa Sokoła-Wysoczańska, Katarzyna Czyż, Anna Wyrostek

**Affiliations:** 1 The Lumina Cordis Foundation, Szymanowskiego 2a, 51-609 Wrocław, Poland; sokola@libero.it; 2 Institute of Animal Breeding, Wrocław University of Environmental and Life Sciences, Chełmońskiego 38c, 51-630 Wrocław, Poland; anna.wyrostek@upwr.edu.pl

**Keywords:** linseed, ethyl esters, fish oil, triglycerides, cholesterol

## Abstract

Dyslipidemia is a serious condition affecting an increasing number of people, and thus, preventive measures, including supplementation, are being developed. We aimed to compare the effect of linseed oil, its ethyl esters and fish oil supplementation on the serum lipid profiles of rats fed a high-fat diet. Wistar rats were divided into nine groups. Four of them were fed a high-fat diet for the whole experiment, four groups were fed a high-fat diet before the supplementation period and then the control one with supplements, and one was fed a control diet without supplements. The whole experiment lasted 12 weeks. A significant reduction in blood triglycerides, total cholesterol and the LDL fraction was noted in supplemented groups compared to the controls, especially in groups supplemented with ethyl esters of linseed oil and linseed oil compared to fish oil groups. The results were also more beneficial in groups where, in addition to supplementation, there was also a diet change from a high-fat diet to a control diet during the supplementation period. We may conclude that supplementation with omega-3 fatty acids, combined with a healthy diet, may be a good way of preventing or alleviating dyslipidemia.

## 1. Introduction

Dyslipidemia is a clinical condition that is characterized by a disturbed blood lipid profile, i.e., increased levels of triglycerides (TGs), total cholesterol (TC) and the low-density lipoprotein (LDL) cholesterol fraction, and concurrently lowered levels of high-density lipoprotein (HDL) cholesterol [1,2]. The development of this condition has been attributed to numerous factors, related both to genetics and lifestyle, and poorly controlled dyslipidemia may be related to the state of chronic inflammation, which consequently leads to a number of disorders such as cardiovascular diseases like hypertension, insulin resistance, diabetes, abdominal obesity or kidney disease [3,4]. Numerous strategies have been developed to control dyslipidemia, and they include changes in lifestyle, e.g., diet, physical exercise or pharmacological means [1]. However, a key role in dyslipidemia management and CVD risk prevention is attributed to nutritional factors [5], and supplementation with omega-3 fatty acids is considered to be one of the most significant approaches in this range. Dietary supplements other than PUFAs (polyunsaturated fatty acids) can be used to prevent dyslipidemia or to support its treatment, and they include, for example, plant sterols or stanols, berberine, red yeast rice, garlic, resveratrol, sour tea, green tea, curcuminoids or anthocyanins [6], policosanol, niacin, tocotrienols, pantethine, gugulipids [7] and probiotics [8].

Omega-3 fatty acids, along with omega-6 ones, belong to the group of polyunsaturated fatty acids (PUFAs). The main representatives of PUFAs are linoleic acid (LA, n-6) and alpha-linolenic acid (ALA, n-3). Both groups of acids, in opposite ways, influence the metabolic functions of the body; i.e., omega-3 plays an anti-inflammatory role, while omega-6 initiates pro-inflammatory reactions [9]. The most important issue in the case of the intake of these acids is the ratio of omega-6 to omega-3, which, according to numerous studies, should be about 4.5:1 to 10:1, while currently, it is much higher, at a level of 15–16:1 or even 20:1 [10,11,12,13].

Linseed oil has been manufactured from linseed for years due to its beneficial effects on human health [14]. The main active component of linseed oil is alpha-linolenic acid from the omega-3 family (ALA), which is the precursor of eicosapentaenoic (EPA) and docosahexaenoic (DHA) acids. Unfortunately, linseed and linseed oil also contain some potentially harmful substances, e.g., cyanogenic compounds, linatine or phytic acid [15]. The solution for this may be supplementation with the ethyl esters of linseed oil [16,17,18]. The esters demonstrate a higher level of bioavailability in relation to the traditional triglyceride form, and their absorption and incorporation into various blood lipid fractions is improved [19], which mainly results from the simple molecular structure and more efficient kinetics of the release of free acid, which ensures more effective digestion. Ethyl esters of linseed oil exhibit very low toxicity, which is comparable to that of natural triglycerides of plant origin, and are free from toxic substances from the group of cyanohydrates (amygdalin, linamarin) contained in the oil. They are also resistant to acidic hydrolysis in the liver environment, which enables their transport to further segments of the gastrointestinal tract. At the same time, they contain the beneficial health components of linseed oil. Furthermore, the oxygen solubility of esters is considerably reduced compared to oil, which leads to increased stability over a longer time. They also exhibit lower susceptibility to oxidation, epoxidation and peroxidation processes compared to the raw material they are produced from (linseed oil) [16,20,21]. An in vitro study on human adipose-derived mesenchymal stem cells also demonstrated the higher biological activity of ethyl esters of linseed oil compared to linseed oil [22]. Another popular source of omega-3 acids is fish oil, which contains mainly EPA and DHA fatty acids; however, some concerns are related to the presence of environmental toxins, like dioxins, mercury and polychlorinated biphenyls, or hypervitaminosis related to high levels of fat-soluble vitamins D and A in fish oil [23,24,25].

Thus, the aim of this study was to examine the effect of the ethyl esters of linseed oil supplementation on the lipid profile of rats’ blood and compare it to the effect of linseed oil and fish oil, which are fatty acid sources commonly used in dietary supplementation. This study also considered various dietary patterns applied in rat feeding. To our knowledge, no such studies on the ethyl esters of linseed oil have been conducted so far.

## 2. Materials and Methods

### 2.1. Animals and Scheme of the Experiment

This study was carried out on monozygotic male Wistar rats from Charles Rivers Laboratories (Sulzfeld, Germany). The animals were maintained individually in the vivarium of the Faculty of Veterinary Medicine, Wrocław University of Environmental and Life Sciences, Poland. The temperature in the room was about 21 °C, and a 12 h light/dark cycle was provided. The animals were randomly divided into 9 groups (n = 8). They were fed with the control diet (C) with w/10% energy from fat (diet No. C 1090-10) and high-fat diet (H) with w/70% energy from fat (42% fat) (diet No. C1090-70) from Altromin International (Germany), as shown in Table 1, and had ad libitum access to water. Prior to the experiment, the rats had a 2-week acclimatization period, followed by a 4-week period when the animals (excluding the control group) were fed a high-fat diet to induce hyperlipidemia. The experiment lasted 8 weeks; during this time, the animals from the experimental groups were supplemented with linseed oil (LO), which was the raw material for the production of ethyl esters, linseed oil ethyl esters (EEs) and fish oil (FO). All supplements were administered orally in the amount of 0.04 g/kg of body weight using a syringe.

This study was carried out with the agreement of the 2nd Local Animal Ethics Committee, Wrocław University of Environmental and Life Sciences, Poland (approval No. 79/2010).

### 2.2. Supplements

The synthesis of the linseed oil ethyl esters was performed using the technology developed at the University of Wrocław (Wrocław, Poland) [26]. The technology for the manufacturing of the ethyl esters and the characteristics of the ethyl esters are presented in the study of Sokoła-Wysoczańska et al. [16]. In brief, the technology consists of the transesterification of linseed oil (a mixture of triglycerides of omega-3, -6, -9 fatty acids) with ethanol in the presence of the catalyst. The first stage of this process involves transesterification in an anaerobic atmosphere, and then unreacted bioethanol is removed from the post-reaction mixture, and the glycerin phase is separated from the raw ester phase in gravity separators. Then, the raw esters are subjected to a cleaning process, first by centrifugation and then using a residual gas alcohol depot with nitrogen, and the residual glycerin phase is precipitated. The glycerin phase is separated in the last step of the process.

For comparative purposes, we also applied raw linseed oil, which was a substrate for the synthesis of ethyl esters, and commercially available fish oil (cod liver). The fatty acid profiles of the supplements applied in this study are presented in Table 2.

### 2.3. Blood Sampling and Analyses

**Figure 1:**
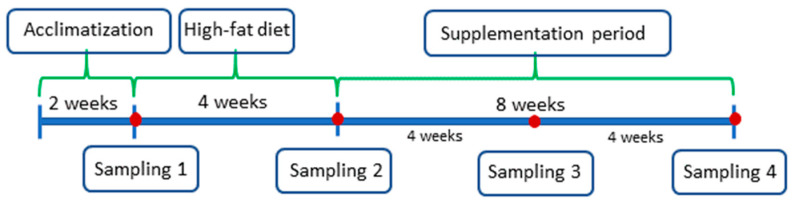
Scheme of sample collection during the experiment.

Blood samples from the experimental rats were collected 4 times in total: before the start of the study—after the acclimatization period (sampling 1); after 4 weeks of high-fat feed—the beginning of supplementation (sampling 2); and twice at 4-week intervals (sampling 3 and 4) (Figure 1). The samples were collected after an overnight fasting. The samples were analyzed for the content of triglycerides, total cholesterol, low-density lipoprotein (LDL) cholesterol and high-density lipoprotein (HDL) cholesterol. These analyses were performed on an A25 automatic biochemical analyzer (Biosystems S.A., Barcelona, Spain) using enzymatic colorimetric assays at the VETLAB veterinary laboratory in Wroclaw, Poland.

### 2.4. Body Weight Control

The body weight of the rats was measured at the beginning and at the end of the experiment using a laboratory scale. Based on the measurements, the weight gains over the period of the experiment were calculated for particular groups.

### 2.5. Statistical Analysis

The results were analyzed statistically using Statistica 13.0 (StatSoft, Krakow, Poland) and presented as mean values and standard deviation (SD). The normality of the distribution of the results was verified using the Shapiro–Wilk test. The results were analyzed using one-way analysis of variance (ANOVA). The significance of differences between the mean values was determined using Tukey’s test at the significance level of *p* < 0.05.

## 3. Results

### 3.1. Body Weight

The body weights determined at the beginning and at the end of the experiment as well as the gains over that period are presented in Table 3.

No significant differences between the groups were found at the beginning of the experiment. On the last day of the experiment, the highest value was found in group FO-H, i.e., the group fed a high-fat diet during the whole experiment and supplemented with fish oil, and it was about 7% higher compared to the LO-H-C and FO-H-C groups, 10% higher compared to the C and LO-H groups, and 12% and 14% higher compared to the EE-H-C and C-H-C groups, respectively. All these differences were confirmed statistically (*p* < 0.05). In turn, the body weight gains over the period of the study proved to be the highest in group EE-H, which was fed a high-fat diet and supplemented with ethyl esters, and it was 21–33% higher compared to the groups fed a high-fat diet for the first 4 weeks, about 29%, 30% and 43% higher than the values for the LO-H, C and C-H-C groups, respectively (*p* < 0.05).

### 3.2. Triglyceride Content

The results of triglyceride content in rats’ blood are presented in Table 4.

An analysis of triglyceride content in the blood of the experimental rats demonstrated no significant differences between the groups in samplings 1 and 2. However, in the case of sampling 3, the lowest value of this parameter was found in group EE-H-C, and it was about 43%, 47% and 38% lower compared to groups C, C-H and C-H-C (*p* < 0.05), respectively, and about 41% and 45% lower compared to groups EE-H and FO-H (*p* < 0.05), respectively. A significant decrease in the value of the triglyceride level was also found for groups LO-H (about 17% compared to C-H, *p* < 0.05) and FO-H (about 32%, 37% and 28% in relation to groups C, C-H and C-H-C, respectively, *p* < 0.05) (Table 4).

In an analysis of the content of triglycerides between the samplings within particular groups, statistically significant differences were noted in group C-H-C between samplings 2 and 3 (*p* < 0.05) and in groups EE-H, LO-H-C, EE-H-C and FO-H-C between samplings 1 and 2 and between samplings 3 and 4 (*p* < 0.05) (Table 4).

### 3.3. Total Cholesterol Content

The content of total cholesterol in particular groups of rats is presented in Table 5.

No significant differences in the content of total cholesterol were observed in the first two samplings. In the third sampling, the lowest value was found in group LO-H-C, and it was about 17%, 25%, 20% and 17% lower compared to groups C, C-H, C-H-C and FO-H-C, respectively (*p* < 0.05). In sampling 4, statistically significant differences were noted between groups C and C-H and all supplemented groups (*p* < 0.05), and between group C-H-C and groups EE-H, LO-H-C and EE-H-C (*p* < 0.05). The lowest value of this parameter was found in group LO-H-C, and it was about 30%, 34% and 26% lower compared to groups C, C-H and C-H-C, respectively (*p* < 0.05) (Table 5).

Considering the total cholesterol level between the samplings in particular groups, no statistically significant differences were observed in the control groups. In the case of group LO-H, differences were noted between samplings 1 and 2 and samplings 3 and 4 (*p* < 0.05); in group EE-H, between samplings 1 and 2 and sampling 4 (*p* < 0.05); in group FO-H, between samplings 1 and 4 and between sampling 2 and sampling 4 (*p* < 0.05). In the case of groups LO-H-C and EE-H-C, differences were confirmed between samplings 1 and 2 and sampling 4, as well as between samplings 2 and 3 (*p* < 0.05); in group FO-H-C, differences were confirmed between samplings 2 and 4 (*p* < 0.05) (Table 5).

### 3.4. Low-Density Lipoprotein Cholesterol Content

The content of the LDL cholesterol fraction in the examined rats’ blood is presented in Table 6.

No statistically significant differences in the case of the LDL cholesterol fraction were noted in samplings 1 and 2. In sampling 3, the lowest value of this parameter was observed in group EE-H-C, and it was about 24%, 33%, 39% and 24% lower compared to groups C, C-H-C, C-H and FO-H, respectively (*p* < 0.05). In sampling 4, the lowest value of the LDL fraction was found in group EE-H-C, and it was about 35%, 35%, 45% and 22% lower compared to groups C, C-H-C, C-H and FO-H-C (*p* < 0.05) (Table 6).

In the case of comparison within the groups, statistically confirmed differences were found between samplings 1 and 2 and samplings 3 and 4 in group C-H (*p* < 0.05); between samplings 1 and 2 and sampling 4 in group EE-H (*p* < 0.05); between samplings 1, 2 and 3 and sampling 4 in group FO-H (*p* < 0.05); and between samplings 1 and 2 and samplings 3 and 4 in group EE-H-C (*p* < 0.05) (Table 6).

### 3.5. High-Density Lipoprotein Cholesterol Content

The content of the HDL cholesterol fraction obtained in this study is presented in Table 7.

Statistically significant differences between the groups in the HDL cholesterol fraction were found starting from the beginning of the experiment, unlike in the case of the other analyzed parameters. In the case of sampling 1, the lowest value of the HDL fraction was noted in group LO-H-C, and it was about 20% lower compared to group FO-H (*p* < 0.05). For sampling 2, the lowest value of this parameter was found in group EE-H-C, and it was about 17%, 24%, 20%, 17% and 18% lower compared to groups C, C-H, C-H-C, EE-H and FO-H, respectively (*p* < 0.05). In the case of sampling 3, in turn, the lowest value was noted in group LO-H, and it was about 19%, 24%, 16%, 23% and 17% lower compared to groups C, C-H, C-H-C, FO-H and LO-H-C, respectively (*p* < 0.05). Finally, in the case of sampling 4, the lowest value of the HDL fraction was observed in group EE-H, and it was about 21% and 16% lower compared to groups C and C-H (*p* < 0.05) (Table 7).

Considering the HDL cholesterol content between the samplings in particular groups, in groups C-H and C-H-C, statistically significant differences were confirmed between sampling 2 and samplings 1, 3 and 4, as well as between samplings 3 and 4 (*p* < 0.05). In groups EE-H and FO-H, differences were observed between samplings 1, 2 and 3 and sampling 4 (*p* < 0.05); in group LO-H-C, between samplings 2 and 3 and sampling 4 (*p* < 0.05); and in group EE-H-C, between samplings 1 and 2 and sampling 4 (*p* < 0.05) (Table 7).

## 4. Discussion

### 4.1. Body Weight

It is obvious that a high-fat diet can negatively affect body weight, leading to overweight or obesity; however, some concerns in this aspect may be also related to diet supplementation with oils both of plant and animal origin. In this study, the body weight and weight gains were higher in the groups fed a high-fat diet for the whole experiment compared to the groups fed a control diet during the supplementation period, which suggests that this feature is affected by diet rather than oils. This is consistent with the results obtained by Majewski et al. [27], who found no significant modification of body weight gains in rats supplemented with fish oil compared to a control group. In turn, comparable body weights were noted by Dias et al. [28] in all groups in a study where rats were fed a control diet, a high-fat diet and a high-fat diet supplemented with linseed oil. A study conducted by Seliem et al. [29] demonstrated a significantly higher body weight at the end of the experiment in rats fed a high-fat diet supplemented with linseed oil compared to a control group, but the body weight of these rats was concurrently significantly lower in relation to a group fed a high-fat diet without supplementation.

An increase in body weight in animals fed a high-fat diet can be attributed to the high energy density of fats, which means there is an increase in energy consumption with increasing fat intake [30]. In this study, rats from groups LO-H and EE-H were characterized by lower body weight gains compared to those in the C-H group, and the opposite was found for the FO-H group. This is consistent with the study performed by Bashir et al. [31], who noted that linseed oil supplementation significantly reduced body weight. Similar results were obtained in a study when fish oil was supplemented [32]. Also, the study conducted by Vijaimohan et al. [33] demonstrated a reduction in liver weight in rats fed a high-fat diet and supplemented with linseed oil, and the authors suggested that the hypolipidemic and antioxidant activities of linseed oil are responsible for its beneficial effects on weight gain. Moreover, it was concluded in another study that the effect of dietary linseed oil on body weight may be attributed to its α-linolenic acid (ALA) content, which reduces adipocyte hypertrophy, levels of inflammatory biomarker proteins, monocyte chemoattractant protein-1 (MCP-1), TNF-α and T-cell infiltration in adipose tissue [34].

### 4.2. Blood Lipid Profile

The consumption of a high-fat diet leads to an increase in serum levels of triglycerides, total cholesterol and the LDL cholesterol fraction in rats compared to animals fed a normal diet [29,35].

Kontostathi et al. [5] conducted a study investigating the effect of various fish oils (sardine, trout, cod liver, eel) on the blood lipid profile of mice fed a high-fat diet before the beginning of supplementation. The authors observed a statistically significant decrease in total cholesterol and HDL levels in all groups and a significant decrease in triglyceride content in the groups supplemented with eel and cod liver oils.

In a study conducted by Gomes et al. [36], healthy rats were supplemented with linseed oil, and the authors observed no significant differences between the control and supplemented groups in the serum content of total cholesterol and triglycerides. In another study, Wistar rats were supplemented with hot- and cold-pressed linseed oil, and both supplements caused a significant decrease in triglycerides, total cholesterol and the LDL fraction, as well as an increase in the HDL level, compared to a control group and a group fed a hypercholesterolemic diet [37].

A study on Wistar rats fed a high-fat diet for 3 weeks before supplementation and another for 3 weeks with supplementation with flaxseed oil and animal-origin omega-3 capsules was conducted by Shahidi et al. [38]. In the case of flaxseed administration, the levels of triglycerides and total cholesterol decreased after the end of the experiment to a value slightly below the baseline; i.e., before a high-fat diet introduction, the level of LDL decreased to a value twice lower, while the HDL level increased twice. Similar results were observed in the group supplemented with omega-3 capsules.

Linseed oil supplementation caused a significant decrease in the level of total cholesterol, triglycerides and LDL and an increase in the HDL level in rats fed a high-fat diet supplemented with linseed oil compared to a high-fat diet; however, these values were still significantly higher than those in a control group [29]. Similar results were obtained by Elimam and Ramadan [39]. The systematic review by Yue et al. [40] on the effect of ALA administration on blood lipid profiles revealed a reduction in triglycerides, total cholesterol and LDL cholesterol content after supplementation and no effect on HDL cholesterol content [40].

It is suggested that the beneficial impact of linseed oil on blood lipid profile is just due to the content of omega-3 acids, as they promote the excretion of cholesterol via bile and thus lead to a depletion of hepatic cholesterol and an enhancement of the synthesis of free cholesterol [29]. Additionally, in the case of plant omega-3 sources, it is suggested that diets rich in alpha-linolenic acid reduce the storage of fat in the liver as ALA promotes fatty acids’ β-oxidation and reduces their synthesis [41]. Omega-3 acids reduce the content of triglycerides due to the regulation of peroxisome proliferator-activated receptor (PPAR) regulating hepatic fatty acid catabolism and sterol regulatory element-binding protein-1 (SREBP-1) regulating hepatic fatty acid synthesis [42]. According to the literature, the suggested effect of omega-3 fatty acids on plasma triglyceride reduction involves an increase in the oxidation of fatty acids that attenuates lipogenesis in the liver and further VLDL formation. However, the exact mechanism responsible for the triglyceride-lowering effect of omega-3 acids is still unclear [43]. It has been also suggested that the mechanism underlying triglyceride level reduction by omega-3 PUFAs may be modified by nuclear receptors. It was demonstrated that these acids are involved in a few pathways leading to gene transcription; they affect receptors modulating triglyceride levels [44,45]. The effect of omega-3 fatty acids on triglyceride metabolism causing its levels to decrease results from a decrease in VLDL particle secretion from the liver and an increase in the degradation of hepatic apolipoprotein B [46]. Studies demonstrated that fish oil supplementation increases the level of HDL, and this effect is suggested to be attributed to a decrease in plasma free fatty acids and further decrease in the transfer of cholesterol free esters from HDL particles to LDL and VLDL particles [47].

Undoubtedly, some differences may be observed between the effects of plant and fish sources of omega-3 fatty acids. Linseed oil and its ethyl esters are a rich source of alpha-linolenic acid (ALA), which is a precursor of other acids from the omega-3 group, i.e., eicosapentaenoic acid (EPA) and docosahexaenoic acid (DHA), which in turn are abundant in fish oil. Supplementation with plant sources of omega-3s provides the substrates for further synthesis of EPA and DHA in an organism [16]. However, the same metabolic pathway is engaged in the conversion of both linoleic acid (LA) and ALA, with the participation of elongases and desaturases. In this process, LA competes with ALA for the enzymes, and an excess of LA can reduce ALA transformation to EPA and DHA, leading to the formation of high amounts of arachidonic acid (AA). Concurrently, the conversion of ALA to EPA is much higher than that of EPA to DHA [48,49,50].

The limitation of the study presented is a relatively low number of animals in particular groups, and presumably an insufficient time of high-fat diet administration before the beginning of supplementation, which caused no very distinct changes in the serum lipid profile to be observed. However, the results may be treated as preliminary ones suggesting the rationale for further, more extensive research in this field.

## 5. Conclusions

All supplements examined in this study caused a reduction in lipid profile indices in supplemented groups compared to the controls. The decrease was more pronounced in groups supplemented with ethyl esters of linseed oil and linseed oil compared to fish oil groups. The results were also more beneficial in groups where, in addition to supplementation, there was also a diet change from a high-fat diet to a control diet during the supplementation period.

## Figures and Tables

**Table 1:** Scheme of the experiment.

	Diet		Supplementation Weeks 5–12
Group	Weeks 1–4	Weeks 5–12	
C	control	control	-
C-H	high-fat	high-fat	-
C-H-C	high-fat	control	-
LO-H	high-fat	high-fat	Linseed oil
EE-H	high-fat	high-fat	Ethyl esters
FO-H	high-fat	high-fat	Fish oil
LO-H-C	high-fat	control	Linseed oil
EE-H-C	high-fat	control	Ethyl esters
FO-H-C	high-fat	control	Fish oil

**Table 2:** Fatty acid profiles of supplements used in this study (% of total fatty acids) [17].

Acid	LO	EEs	FO
palmitic acid (C16:0)	4.37	4.44	11.36
stearic acid (C18:0)	3.79	3.43	2.68
oleic acid (C18:1)	16.41	16.73	23.95
linoleic acid (C18:2)	16.24	16.68	1.43
linolenic acid (C18:3)	56.29	58.71	−
eicosapentaenoic acid (C20:5)	−	−	8.13
docosahexaenoic acid (C22:6)	−	−	9.87

LO—linseed oil; EEs—linseed oil ethyl esters; FO—fish oil.

**Table 3:** Body weight of the experimental animals at the beginning and end of the experiment (mean ± SD).

Group	Body Weight (g)		
	Beginning	Final	Gains
C	380.50 ± 9.23	576.13 ± 29.57 ^c,d^	195.63 ± 23.05 ^c^
C-H	383.75 ± 16.59	627.13 ± 36.91 ^a,b^	243.38 ± 38.57 ^a,b^
C-H-C	380.00 ± 15.32	556.88 ± 24.47 ^d^	176.88 ± 27.37 ^c^
LO-H	382.88 ± 27.30	579.50 ± 57.36 ^c,d^	196.63 ± 60.78 ^c^
EE-H	377.25 ± 17.49	599.38 ± 27.93 ^a,b,c^	222.13 ± 41.41 ^a,b,c^
FO-H	381.50 ± 16.20	634.88 ± 38.51 ^a^	253.38 ± 45.37 ^a^
LO-H-C	382.34 ± 19.10	591.25 ± 47.28 ^b,c,d^	208.91 ± 37.91 ^b,c^
EE-H-C	379.38 ± 12.70	569.38 ± 26.31 ^c,d^	190.00 ± 33.31 ^c^
FO-H-C	384.38 ± 14.24	593.75 ± 30.23 ^b,c,d^	209.38 ± 42.37 ^b,c^

^a–d^ Different letters within the columns indicate statistical differences between the groups at *p* < 0.05.

**Table 4:** Triglyceride content in rats’ blood (mean ± SD).

Group	Triglycerides (nmol/L)			
	Sampling 1	Sampling 2	Sampling 3	Sampling 4
C	3.47 ± 0.37	3.45 ± 0.27	3.35 ± 0.25 ^a,b^	3.39 ± 0.45 ^a,b^
C-H	3.46 ± 0.70	3.61 ± 0.27	3.59 ± 0.33 ^a^	3.66 ± 0.45 ^a^
C-H-C	3.36 ± 0.29	3.55 ± 0.27 *	3.12± 0.40 ^b,#^	3.25 ± 0.27 ^a,b^
LO-H	3.57 ± 0.74	3.62 ± 0.54	3.24 ± 0.29 ^a,b^	3.05 ± 0.51 ^b^
EE-H	3.24 ± 0.61 *	3.47± 0.57 *	2.69 ± 0.41 ^c,#^	2.31 ± 0.50 ^c,d,#^
FO-H	3.39 ± 0.81	3.52 ± 0.68	3.47 ± 0.52 ^a,b^	3.35 ± 0.62 ^a,b^
LO-H-C	3.32 ± 0.73 *	3.49 ± 0.45 *	2.59 ± 0.56 ^c,d,#^	2.28 ± 0.23 ^c,d,#^
EE-H-C	3.46 ± 0.50 *	3.51 ± 0.65 *	1.92 ± 0.35 ^c,e,#^	1.87 ± 0.39 ^d,#^
FO-H-C	3.37 ± 0.63 *	3.53 ± 0.40 *	2.36 ± 0.40 ^d,#^	2.59 ± 0.44 ^c,#^

^a–e^ Different letters within the columns indicate statistical differences between the groups at *p* < 0.05. *^,#^ Different symbols within the rows indicate statistical differences between the samplings at *p* < 0.05.

**Table 5:** Total cholesterol content in rats’ blood (mean ± SD).

Group	Total Cholesterol (nmol/L)			
	Sampling 1	Sampling 2	Sampling 3	Sampling 4
C	2.80 ± 0.31	2.69 ± 0.38	2.76 ± 0.39 ^a,b^	2.79 ± 0.35 ^a^
C-H	2.86 ± 0.31	3.04 ± 0.36	3.05 ± 0.38 ^a^	2.96 ± 0.48 ^a^
C-H-C	2.68 ± 0.29	2.97 ± 0.31	2.85 ± 0.29 ^a,b^	2.65 ± 0.27 ^a,b^
LO-H	2.91 ± 0.27 *	2.99 ± 0.39 *	2.51 ± 0.26 ^b,c,#^	2.29 ± 0.23 ^b,c,#^
EE-H	2.72 ± 0.40 *^,#^	3.01 ± 0.41 *	2.57 ± 0.37 ^b,c,#^	2.13 ± 0.30 ^c,$^
FO-H	2.87 ± 0.26 *^,#^	3.03 ± 0.38 *	2.64 ± 0.30 ^b,c,#,$^	2.35 ± 0.32 ^b,c,$^
LO-H-C	2.59 ± 0.29 *^,#^	2.94 ± 0.50 *	2.28 ± 0.34 ^c,#,$^	1.96 ± 0.33 ^c,$^
EE-H-C	2.66 ± 0.36 *^,#^	2.96 ± 0.33 *	2.35 ± 0.37 ^c,#,$^	2.08 ± 0.40 ^c,$^
FO-H-C	2.64 ± 0.62	3.00 ± 0.44 *	2.75 ± 0.30 ^a,b^	2.28 ± 0.45 ^b,c,#^

^a–c^ Different letters within the columns indicate statistical differences between the groups at *p* < 0.05. *^,#,$^ Different symbols within the rows indicate statistical differences between the samplings at *p* < 0.05.

**Table 6:** LDL cholesterol content in rats’ blood (mean ± SD).

Group	LDL Cholesterol (nmol/L)			
	Sampling 1	Sampling 2	Sampling 3	Sampling 4
C	0.74 ± 0.07	0.71 ± 0.10	0.74 ± 0.14 ^b,c^	0.79 ± 0.13 ^b^
C-H	0.73 ± 0.12 *	0.79 ± 0.10 *	0.92 ± 0.14 ^a,#^	0.93 ± 0.14 ^a,#^
C-H-C	0.75 ± 0.17	0.78 ± 0.10	0.84 ± 0.15 ^a,b^	0.79 ± 0.12 ^b^
LO-H	0.69 ± 0.17	0.70 ± 0.19	0.63 ± 0.12 ^c,d^	0.58 ± 0.07 ^c,d^
EE-H	0.71 ± 0.11 *	0.68 ± 0.07 *	0.61 ±0.16 ^c,d,^*^,#^	0.53 ± 0.13 ^c,d,#^
FO-H	0.73 ± 0.09 *	0.69 ± 0.10 *^,#^	0.74 ± 0.13 ^b,c,^*	0.59 ± 0.08 ^c,d,#^
LO-H-C	0.68 ± 0.11	0.69 ± 0.11	0.58 ± 0.16 ^c,d^	0.56 ± 0.10 ^c,d^
EE-H-C	0.74 ± 0.17 *	0.72 ± 0.14 *	0.56 ± 0.13 ^d,#^	0.51 ± 0.11 ^d,#^
FO-H-C	0.71 ± 0.16	0.69 ± 0.09	0.72 ± 0.17 ^b,c,d^	0.65 ± 0.14 ^a^

^a–d^ Different letters within the columns indicate statistical differences between the groups at *p* < 0.05. *^,#^ Different symbols within the rows indicate statistical differences between the samplings at *p* < 0.05.

**Table 7:** HDL cholesterol content in rats’ blood (mean ± SD).

Group	HDL Cholesterol (nmol/L)			
	Sampling 1	Sampling 2	Sampling 3	Sampling 4
C	1.48 ± 0.21	1.64 ± 0.22 ^a,b^	1.51 ± 0.19 ^a,b^	1.43 ± 0.18 ^a^
C-H	1.45 ± 0.21 ^#,$^	1.80 ± 0.16 ^a,^*	1.61 ± 0.12 ^a,#^	1.35 ± 0.23 ^a,b,$^
C-H-C	1.36 ± 0.11 ^#,$^	1.71 ± 0.19 ^a,^*	1.46 ± 0.21 ^a,b,c,#^	1.26 ± 0.16 ^a,b,c,$^
LO-H	1.46 ± 0.46	1.40 ± 0.22 ^b,c^	1.23 ± 0.24 ^d^	1.23 ± 0.16 ^a,b,c^
EE-H	1.56 ± 0.31 *	1.64 ± 0.28 ^a,b,^*	1.43 ± 0.27 ^a,b,c,d,^*	1.13 ± 0.11 ^c,#^
FO-H	1.62 ± 0.23 ^a,^*	1.65 ± 0.28 ^a,b,^*	1.59 ± 0.23 ^a,^*	1.26 ± 0.19 ^a,b,c,#^
LO-H-C	1.29 ± 0.20 ^b,^*^,#^	1.44 ± 0.18 ^b,c,^*	1.48 ± 0.17 ^a,b,c,^*	1.21 ± 0.18 ^b,c,#^
EE-H-C	1.40 ± 0.21 *	1.36 ± 0.23 ^c,^*	1.27 ± 0.08 ^c,d,^*^,#^	1.14 ± 0.26 ^c,#^
FO-H-C	1.39 ± 0.30	1.39 ± 0.27 ^b,c^	1.35 ± 0.19 ^b,c,d^	1.28 ± 0.07 ^a,b,c^

^a–d^ Different letters within the columns indicate statistical differences between the groups at *p* < 0.05. *^,#,$^ Different symbols within the rows indicate statistical differences between the samplings at *p* < 0.05.

## Data Availability

Data is contained within the article.
